# MACC1 and MET as markers associated with progression and metastasis in cutaneous melanoma

**DOI:** 10.3389/fonc.2023.1146092

**Published:** 2023-07-10

**Authors:** Yan Zhou, Cody J. Rasner, Alessio Giubellino

**Affiliations:** ^1^ Department of Laboratory Medicine and Pathology, University of Minnesota, Minneapolis, MN, United States; ^2^ Department of Laboratory Medicine and Pathology, University of Minnesota Medical School, Minneapolis, MN, United States; ^3^ Masonic Cancer Center, University of Minnesota, Minneapolis, MN, United States

**Keywords:** MACC1, MET, melanoma, metastasis, biomarker

## Abstract

Cutaneous melanoma is the most common lethal malignancy among skin cancers and has a high propensity for metastasis. Understanding the mechanisms governing tumorigenesis, progression and metastasis as well as identifying biomarkers guiding risk stratification and management of the disease is essential. MACC1 has been found to play key roles in cancer cell migration, invasion, epithelial-to-mesenchymal transition, and metastasis in various types of cancer, through activation of MET signaling. In this study, we examined the extent of MACC1 and MET protein expression by immunohistochemical staining in a tissue microarray constructed from twenty-three melanomas and ten melanocytic nevi. We observed significantly higher levels of MACC1 expression on average in metastatic melanomas, comparing to primary melanomas and nevi. MET expression in metastatic melanomas was also significantly higher than in nevi. MACC1 expression does not appear to correlate with MET expression in nevi and primary melanomas. However, this correlation appears stronger in metastatic melanomas, where seven (78%) of nine cases show intermediate to high expression of both MACC1 and MET. The expressions of MACC1 and MET do not show significant differences based on other clinicopathologic factors including patient age, gender, histologic subtypes, depth of invasion, and staging. Our study suggests that high expression of MACC1 or both MACC1 and MET is associated with metastasis of cutaneous melanoma.

## Introduction

Cutaneous melanoma is the fourth and fifth most frequently diagnosed cancer in the United States in women and men, respectively, and the most common lethal cancer among all skin malignancies (1). Continued elucidation of the pathogenesis of melanoma is critical in abating the associated mortality and morbidity. Biomarkers that can be promptly assessed in melanoma patient specimens are of great interest for guiding appropriate diagnosis and management and evaluating the prognosis (2–4).

Recently, the critical roles of *MACC1* (metastasis-associated in colon cancer-1) in cancer progression, invasion and metastasis has been recognized in colon cancer and various other types of cancer (5–8). MACC1 is found to be a key transcription factor in the process of epithelial-to-mesenchymal transition (6, 7, 9). High expression of MACC1 protein in colorectal cancer or high MACC1 transcripts in the blood are strongly associated with advanced disease with metastasis, making it a potentially useful biomarker (10–12).

Mechanistically, *MACC1* encodes a key regulatory protein involved in hepatocyte growth factor (HGF)/MET signaling activation (13, 14). MET, a receptor tyrosine kinase also known as hepatocyte growth factor receptor, is encoded by the proto-oncogene *MET*. HGF/MET signaling through ligand-dependent autocrine or paracrine mechanisms promotes several critical steps in cancer cell invasion and metastasis, such as cell scattering, migration, extracellular matrix degradation, and angiogenesis (15). HGF/MET signaling has been recognized to have an etiopathogenetic role in melanoma progression and metastasis, as well as immune microenvironment modulation and therapeutic resistance (16). Given the mechanistic interaction between *MACC1* and *MET*, it is logical to postulate roles of *MACC1* in melanoma progression and metastasis. Prior studies demonstrated that knockdown of *MACC1* significantly inhibited the proliferation, migration, and invasion capability of melanoma cells *in vitro* (17). However, there are limited studies in the current literature describing the expression of MACC1 in various stages of cutaneous melanoma in patient specimens or elucidating the role of MACC1 as well as its functional correlation with *MET* in tumorigenesis, progression, and metastasis of melanoma.

In the present study, we aim to investigate MACC1 and MET protein expression in melanocytic nevi, primary and metastatic human melanoma samples using immunohistochemistry, and further evaluating the prognostic value of MACC1 and MET, as well as their potential as biomarkers in melanoma.

## Methods

### Case selection and tissue microarray construction

Following IRB approval, cases with the diagnoses of cutaneous melanoma (primary and metastatic) from 2009 to 2019 were searched within the University of Minnesota pathology database. Twenty three cases with slides available for review and formalin-fixed paraffin-embedded (FFPE) tissue blocks available for tissue microarray (TMA) construction were selected. Ten age-matched cases with the diagnosis of intradermal nevus were also selected as benign controls. For TMA construction, each melanoma or nevus case was represented by two tissue cores (4µm in thickness). The tissue cores were randomly built with orientation cores into the TMA. The histology of all the foci in the TMA were reassessed and confirmed by two pathologists (AG and YZ).

### Immunohistochemical staining

TMA sections (4 µm) were immunohistochemically stained with MACC1 and MET antibodies using standard methods. Briefly, the sections were de-paraffinized and rehydrated. For antigen retrieval, slides were incubated in 6.0 pH buffer in a steamer for 30 min at 95-98°C, followed by a 20 min cool down period. Subsequent steps were automated using an IHC staining platform (Intellipath, Biocare). Endogenous peroxidase activity was quenched by slide immersion in 3% hydrogen peroxide solution (Peroxidazed, Biocare) for 10 min followed by TBST rinse. A serum-free blocking solution (Background Sniper, (Biocare Medical, Concord, CA) was placed on sections for 10 min. Blocking solution was removed and slides were incubated in primary antibody diluted in 10% blocking solution/90% TBS. Rabbit polyclonal anti-MACC1 (HPA020081) (Sigma, 1:1000) and Rabbit monoclonal anti-MET (clone D1C2 XP(R) (Cell Signaling, 1:50) were incubated for 60 min at room temperature followed by TBST rinse and detection with Novocastra Novolink Polymer Kit (Leica Microsystems Inc., Buffalo Grove, IL) using the manufacturer’s specifications. All slides then proceeded with TBST rinse and detection with diaminobenzidine (DAB) (Biolegend, Dedham, MA). Slides were incubated for 5 min followed by TBS rinse then counterstained with CAT Hematoxylin (Biocare, Concord, CA) for 5 min. Slides were dehydrated and coverslipped.

### Grading of immunohistochemical staining

**Figure 1:**
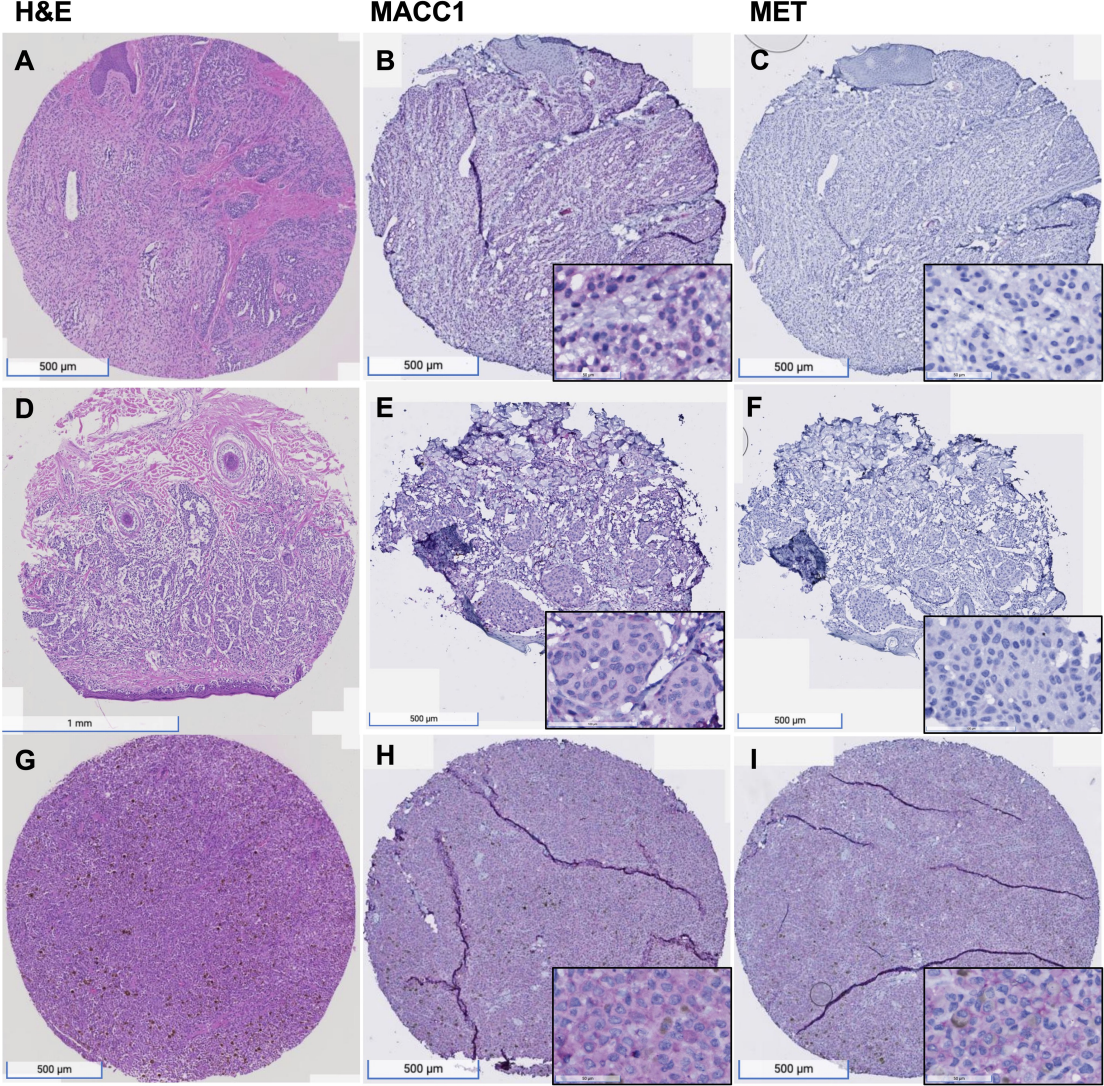
MACC1 and Met expression in melanocytic nevus, primary melanoma and metastatic melanoma. **(A–C)**, An example of nevus, showing intermediate level of MACC1 expression **(B)** and low level of Met expression **(C)**; **(D–F)**, an example of primary melanoma, showing intermediate level of MACC1 expression **(E)** and low level of Met expression **(F)**; **(G–I)**, an example of metastatic melanoma, showing high level of MACC1 expression **(H)** and Met expression **(I)**. Magnification: 20x (insets in B, C, E, F, H, I 200x).

The grading of MET and MACC1 staining of the melanocytic lesions were modified from the H-score method used in prior literature (Toyama et al., 2019). Cytoplasmic staining of MACC1 and MET of at least 5% tumor cells was considered positive (18, 19). The results were reviewed independently by two pathologists (AG and YZ). MACC1 and MET expression was scored to account for both the intensity and extent of the staining of melanocytic cells. The intensity was graded as negative (0), weak (1+), and strong (2+). Representative images of weak and strong expressions of MACC1 and MET proteins are shown in **Figures 1A–I**. The overall MACC1 or MET expression score of each core in the TMA was derived from the sum of each of the intensity values multiplied by the percentage of positive tumor cells (2 × x% + 1 × y% = total score) to equal a range of 0–200. The average score of the two foci in the TMAs from the same component was calculated for analysis. The expression scores were subsequently arbitrarily categorized into low (≤50), intermediate (51-150) and high (151-200) groups.

### Statistical analyses

The mean values of the scores of MACC1 and MET expression in melanoma and nevi were analyzed by student’s t-test or one-way analysis of variance with *post hoc* Tukey test. The associations of MACC1 and MET expression with clinicopathologic factors were analyzed by the χ2 test or Fisher exact test. The correlation between MACC1 and MET expression was evaluated by linear regression model using Graphpad software. P < 0.05 was considered statistically significant.

## Results

### Clinicopathologic features

The demographics, clinicopathologic characteristics, and survival of the twenty-three patients with cutaneous melanomas are summarized in **Table 1**. There are fourteen primary and nine metastatic melanoma cases, covering a range of anatomic locations, depth of invasion and clinical stages. The ages at the time of diagnosis of melanomas range from 20 years to 89 years, with an average age of 60.2 years. Of the twenty-three melanoma patients, nine were male and fourteen were female. Melanomas were located in the head and neck region (n=12), upper and lower extremities (n=3 and 2, respectively), trunk (n=4), as well as two other anatomic locations, vaginal vault and spleen. The ten patients with melanocytic nevi, including four men and six women, range from the age of 20 years to 75 years, with an average age of 49.5 years. Nevi were located in the head and neck region (n=6), lower extremity (n=1) and trunk (n=3). The histologic subtypes of the primary melanomas are either superficial spreading or nodular for the cases in our cohort (**Table 1**). The depth of invasion of the primary melanomas range from 0.5 mm to 10 mm. Clinical stages from I to IV were all represented, and all the metastatic melanoma cases were at clinical stage IV at the time of sample collection. Patient survival data were available in twenty patients with a follow-up period ranging from six months to twelve years (average of sixty-one months, median of forty-eight months). Eight patients were alive, including six with primary melanoma and two with metastatic melanoma. Twelve patients were deceased, including six with primary melanoma and six with metastatic melanoma. Among the deceased patients, the cause of death was not found in three patients based on review of clinical history and one patient died due to a disease other than melanoma.

**Table 1:** Clinicopathologic characteristics of patients with melanoma and intradermal nevus.

Characteristics		Melanoma (N=23)	Nevus (N=10)
Age (year)			
	Average±SD (range)	60.2±18.8 (20-89)	49.5±16.6 (20-75)
	>60	10	3
	<=60	13	7
Gender			
	Male	9	4
	Female	14	6
Location*			
	Head and neck	12 (2)	6
	Upper extremities	3 (1)	0
	Lower extremities	2 (1)	1
	Trunk	4 (4)	3
	Other^#^	2 (1)	0
Subtype			–
Primary	Superficial spreading	8	
	Nodular	6	
Metastatic		9	
Depth of invasion (primary)			–
	<=1 mm	3	
	>1 mm and <=2 mm	6	
	>2 mm and >=4 mm	3	
	>4 mm	2	
LN metastasis at the time of resection			–
	No	11	
	Yes	4	
	Information not available	8	
Clinical stage			–
	I	3	
	II	7	
	III	4	
	IV	9	
Survival			–
	Alive	8	
	Deceased	12	
	Lost for follow up	3	

*The number of metastatic melanomas cases at the indicated locations were listed in the bracket.

#Other locations were vaginal vault and spleen (metastatic lesion).

### MACC1 and MET expressions show an increasing trend among benign nevi, primary melanomas and metastatic melanomas

**Figure 2:**
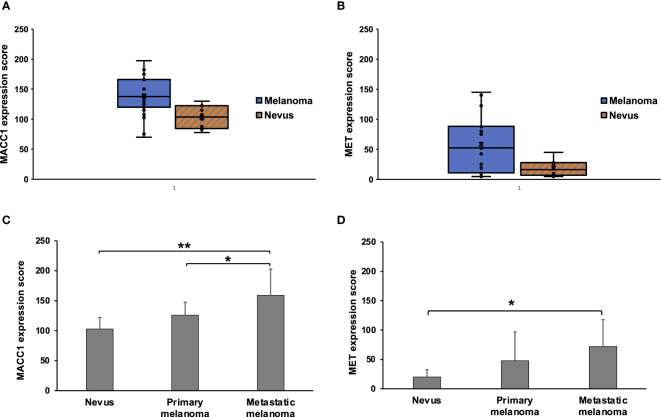
Expression scores of MACC1 and Met in melanocytic nevi and melanomas. **(A, B)**, Box and Whisker plots of MACC1 scores **(A)** and Met scores **(B)** of all the nevus and melanoma cases. **(C, D)**, the average scores and standard deviations of the MACC1 **(C)** and Met expression **(D)** in nevi, primary melanomas and metastatic melanomas (**, p<0.01; *, p<0.05).

MACC1 expression is observed in both melanomas and benign nevi. Specifically, MACC1 is relatively diffusely positive in a cytoplasmic staining pattern in the melanoma and in benign melanocytic cells, but with variable intensity. The average score for MACC1 expression in melanoma is 138.8, ranging from 70 to 200, with a standard deviation of 35.3. The average score for MACC1 expression in nevi is 103, ranging from 77.5 to 130, with a standard deviation of 19.1. The MACC1 expression is significantly higher in melanomas than in nevi (p=0.005) (**Figure 2A**). MET expression in melanoma cases ranges widely from score of 5 to 145, with an average score of 57.7 and standard deviation of 48.1. MET in nevi cases shows negative to weak focal positivity, with an average score of 18.5 (range from 5 to 45) and a standard deviation of 12.4. Similar to MACC1, MET expression in melanomas is significantly higher than in nevi (p=0.018) (**Figure 2B**).

We then evaluated associations between MACC1 or MET expressions and tumor progression. There is a stepwise increase in MACC1 expression in nevi, primary melanomas and metastatic melanomas, with an average score of 103, 125.9, and 159.0, respectively (**Figure 2C**). Metastatic melanomas demonstrate significantly stronger expression of MACC1 than primary melanomas (p=0.032) and nevi (p=0.0003) (**Figure 2C**). While there is a trend in higher MACC1 expression in primary melanomas compared with benign nevi, this difference does not reach statistical significance (p=0.17). The average MET expression shows a progressive increase from nevi to primary melanomas and metastatic melanomas, with an average score of 18.5, 47.9 and 71.9, respectively (**Figure 2D**). The magnitudes of the differences in the average scores of MET expression among these lesions in our cohort appear to be significant, especially between the average scores of nevi and primary melanomas (2.6 folds increase in primary melanomas). However, due to the wide variations among individual cases (e.g. scores ranging from 5 to 142.5 in primary melanomas) and overlaps of the expression levels between different case groups, the difference in MET expression between nevi and primary melanomas does not reach statistical significance (p=0.27). Similar findings are observed in the comparison between primary and metastatic melanomas (p=0.37) (**Figure 2D**). MET expression in metastatic melanomas is significantly higher than in nevi (p=0.017). In addition, a higher proportion of metastatic melanomas demonstrates intermediate to high MET expression (score>50) than primary melanomas (67% vs. 38%), although this trend does not reach statistical significance in our cohort (p=0.387).

Interestingly, MET expression, but not MACC1, appear to be associated with patient survival. When comparing the average scores of MET expression between patients that were alive versus deceased (excluding the cases with unknown cause of death or cause other than melanoma) upon follow-up, MET expression is significantly higher in the deceased group (72.8 in deceased versus 23.0 in alive patients, p=0.01). Of the eight alive patients, one sample had MET expression score higher than 50; while of the eight patients deceased due to melanoma progression, six had MET expression higher than 50 (p=0.01). In contrast, the average scores of MACC1 expression are 131.6 and 162.9 in the alive and deceased patient groups (p=0.06). The expressions of MACC1 and MET do not show significant differences based on patient age, gender, histologic subtypes of the primary melanoma, depth of invasion, and staging.

### Intermediate to high MACC1 and MET expression in the majority of metastatic melanomas

**Figure 3:**
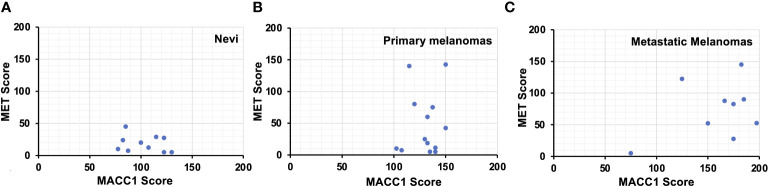
Scatter plots showing the MACC1 scores (X-axis) and Met scores (Y-axis) of individual melanocytic nevus **(A)**, primary melanoma **(B)** and metastatic melanoma cases **(C)**.

It has been postulated that the interaction between MACC1 and MET signaling may be one of the mechanisms contributing to progression and metastasis of melanoma and other malignancies. Therefore, we evaluated if there is a correlation between MACC1 and MET expression in benign nevi, primary and metastatic melanomas. When we performed this analysis, we found no significant quantitatively linear correlation between MACC1 and MET expression levels in nevi (R^2 = ^0.062), primary (R^2 = ^0.02), and metastatic melanomas (R^2 = ^0.14) (**Figure 3**). For nevi, all the ten cases demonstrate negative to variably low levels of MET expression, while express intermediate levels of MACC1 expression. In primary melanomas, MET expression varies widely from scores of 5 to 142.5 with eight (62%) cases showing a relatively low expression, while MACC1 expression for all the cases is at intermediate level, with scores concentrated between 102.5 to 150. On the other hand, seven (78%) of nine metastatic melanomas show intermediate to high expression of both MACC1 and MET. Interestingly, while not statistically significant, MET expression trends up as MACC1 expression increases.

## Discussion

Cutaneous melanoma is a highly aggressive malignancy with high propensity for metastasis and high mortality. Identification of biomarkers is essential in risk stratification and guiding the management. In this pilot study with relatively limited number of cases available, we examined MACC1 and MET protein expression in FFPE tissue samples from patients with benign melanocytic nevi, primary melanomas and metastatic melanomas, and further explored the association between MACC1 and MET expression in these lesions. We observed intermediate to high cytoplasmic expression of MACC1 and low to intermediate expression of MET in both melanocytic nevi and melanomas. The increasing trends of the average MACC1 and MET expression scores may suggest their role in melanoma development, progression and metastasis. Specifically, MACC1 expression showed a statistically significant increase between primary and metastatic melanomas, suggesting MACC1 to be a more sensitive and reliable marker associated with metastasis in cutaneous melanomas. Furthermore, analyses of the correlation between MACC1 and MET expression suggest that coexpression of intermediate to high levels of MACC1 and MET may be associated with metastasis of cutaneous melanoma, whereas the nonparallel expression of MACC1 and MET in benign nevi and primary melanomas suggests that the interplay between MACC1 and MET signaling might not be involved in the benign melanocytic lesions or early stages of melanomas. This study suggests the potential values of MACC1 and MET as useful biomarkers and warrants further evaluation on larger number of melanoma cases.

The role of HGF/MET signaling and expression of MET in melanoma has been studied more extensively than MACC1. Prior studies using melanoma cell lines or patient FFPE tissue demonstrated a wide range of MET expression in both nevi and melanomas. There is a significantly higher proportion and expression level in melanomas than in nevi, while the expression levels are comparable in primary and metastatic melanomas (3, 20). MET expression data in our study is consistent with prior studies in the literature.

Significantly upregulated MACC1 expression has been reported in a number of malignancies including colorectal, liver and lung compared to precursor lesions or normal tissue (5, 21, 22). Studies on MACC1 expression and its functional roles in melanoma are sparse, despite the known mechanistic link between MACC1 and HGF/MET signaling and their critical roles in inducing metastasis. Here we demonstrate elevated level of MACC1 expression in cutaneous melanoma compared to benign melanocytic nevi, as seen in many other cancers. Moreover, MACC1 is associated with metastasis of melanoma. The stepwise increase in the expression of MACC1, in particular, among melanocytic nevi, primary and metastatic melanomas suggest a stronger association of MACC1 with melanoma progression and metastasis. Some studies in other cancer types indicate the prognostic role of MACC1 indicating poorer prognosis or recurrence of disease, however, our study shows that MACC1 is not associated with patient survival in melanoma. Interestingly, higher levels of MET expression, although not statistically distinguishable between primary and metastatic melanomas, is associated with decreased patient survival in our study. Despite the fact that MET expression levels were not significantly different between primary and metastatic melanomas or among cases with different clinical stages, it should be noted though that patients with decreased survival in our cohort skewed towards those with higher clinical stages or metastasis. The possibility of the confounding effect in the analyses cannot be entirely excluded. Serum soluble MET as a prognostic biomarker has been evaluated in patients with metastatic uveal melanoma with promising results showing an association of high-level soluble MET with lower median survival time (23). Prior studies have shown controversial results in the association of MET expression or activating mutations in the *MET* gene with prognosis (16, 24, 25). The inconsistency might be due to the different end points evaluated, technical methods of detection, sample size or other factors. Future study with larger size of patient samples with well documented follow up data may help to further refine these findings.

The roles of MACC1 in cancer invasion, epithelial-to-mesenchymal transition, HGF-triggered scattering of cancer cells and metastasis have been first identified in colorectal cancer *in vitro* and *in vivo*, and subsequently described in other types of cancers (5). To be noted, parallelly strong coexpression of MACC1 and MET was found in colorectal adenocarcinoma with distant metastasis, which was not seen in early stage of colorectal adenocarcinoma (26). We also found coexpression of relatively high levels of MACC1 and MET in the majority of metastatic melanoma cases. This pattern was also seen in a few (38%) primary melanoma cases, however, the correlation between the expression scores of MACC1 and MET is stronger in the metastatic melanomas than in primary melanomas. This correlation is not statistically significant in our study, possibly due to a wide variation of MET expression and limited sample size.

We acknowledge several limitations of the study. First, while we included comparable numbers of primary melanoma and metastatic melanoma cases at various stages, with benign nevi as a control, no melanoma *in situ* cases were included in this study. Given the stepwise increasing trends of MACC1 and MET expression from benign nevi, to primary to metastatic melanomas, it would also be of interest to examine their expressions in melanomas *in situ*, which might provide further suggestions on at which cancer development step(s) MACC1 and MET play a role. Secondly, the MACC1 and MET expression levels varies both within the same lesion and among patients. A larger sample size in future studies should be attempted to increase the statistical power. Thirdly, although our observations are informative in suggesting the critical roles of MACC1 and MET in progression and metastasis of cutaneous melanomas, our study indicates the association of MACC1 and MET expression with disease progression, rather than confirming a causative role of the activation of these genes.

In summary, our data suggest that MACC1 may be a potentially useful biomarker associated with progression of invasion and metastasis in cutaneous melanoma, especially in conjunction with high expression of MET protein.

## Data availability statement

The raw data supporting the conclusions of this article will be made available by the authors, without undue reservation.

## Ethics statement

The studies involving human participants were reviewed and approved by Institutional Review Board, Office of the Vice President for Research, University of Minnesota. Written informed consent for participation was not required for this study in accordance with the national legislation and the institutional requirements.

## Author contributions

YZ and AG contributed to conception and design of the study. YZ, CR and AG performed the slide review, image analyses and scoring of the MACC1 and MET expression. YZ performed the statistical analyses. All authors read, edited, revised, and approve the submitted version of the manuscript. All authors contributed to the article and approved the submitted version.
